# Purification, characterization, cytotoxicity and anticancer activities of L-asparaginase, anti-colon cancer protein, from the newly isolated alkaliphilic *Streptomyces fradiae* NEAE-82

**DOI:** 10.1038/srep32926

**Published:** 2016-09-08

**Authors:** Noura El-Ahmady El-Naggar, Sahar F. Deraz, Hoda M. Soliman, Nehal M. El-Deeb, Sara M. El-Ewasy

**Affiliations:** 1 Department of Bioprocess Development, Genetic Engineering and Biotechnology Research Institute, City of Scientific Research and Technological Applications, Alexandria, Egypt; 2 Department of Protein Research, Genetic Engineering and Biotechnology Research Institute, City of Scientific Research & Technological Applications, Alexandria, Egypt; 3 Department of Botany, Faculty of Science, Mansoura University, Mansoura, Egypt; 4 Biopharmacetical Product Research Department, Genetic Engineering and Biotechnology Research Institute, City of Scientific Research and Technological Applications, Alexandria, Egypt

## Abstract

L-asparaginase is an important enzyme as therapeutic agents used in combination with other drugs in the treatment of acute lymphoblastic leukemia. A newly isolated actinomycetes strain, *Streptomyces* sp. NEAE-82, was potentially producing extracellular L-asparaginase, it was identified as *Streptomyces fradiae* NEAE-82, sequencing product was deposited in the GenBank database under accession number KJ467538. L-asparaginase was purified from the crude enzyme using ammonium sulfate precipitation, dialysis and ion exchange chromatography using DEAE Sepharose CL-6B. Further the kinetic studies of purified enzyme were carried out. The optimum pH, temperature and incubation time for maximum L-asparaginase activity were found to be 8.5, 40 °C and 30 min, respectively. The optimum substrate concentration was found to be 0.06 M. The Km and Vmax of the enzyme were 0.01007 M and 95.08 Uml^−1^min^−1^, respectively. The half-life time (T_1/2_) was 184.91 min at 50 °С, while being 179.53 min at 60 °С. The molecular weight of the subunits of L-asparaginase was found to be approximately 53 kDa by SDS–PAGE analysis. The purified L-asparaginase showed a final specific activity of 30.636 U/mg protein and was purified 3.338-fold. The present work for the first time reported more information in the production, purification and characterization of L-asparaginase produced by newly isolated actinomycetes *Streptomyces fradiae* NEAE-82.

L-asparaginase (L-asparagine aminohydrolase, E.C. 3.5.1.1) is the drug of choice used in combination therapy with other drugs in the treatment of acute lymphoblastic leukemia chemotherapy in children^1^. The demand for L-asparaginase will increase several fold in coming years due to its potential industrial applications as food processing aid in addition to its clinical applications^2^. L-asparaginase is highlighted as a key drug in the treatment of extranodal NK/T-cell lymphoma^3^.

Purification of a protein is an important step for characterization of its physical and biological properties. Moreover, for effective therapeutic use of a protein, it must be free of any contaminants and impurities. However, clinical employments of L-asparaginase are accompanied with fatal allergenic reactions to the patients^4^. These effects are mainly due to L-asparaginase associated L-glutaminase activity and bacterial endotoxins in enzyme preparations^5^. Several research groups have studied L-asparaginase production and purification in attempt to minimize impurities that produce allergenic reactions. The L-asparaginase enzyme was purified from *Penicillium* sp. that was grown on submerged fermentation. Different purification steps including salt precipitation, followed by separation on sephadex G-100-120 gel filtration and DEAE to obtain pure enzyme preparation. The purified enzyme showed 13.97 IU/mg specific activity. The polyacrylmide gel electrophoresis of the pure enzyme exhibited one protein of 66 kDa. The enzyme showed maximum activity at 7.0 pH and 37 °C and *K*m value 4 × 10^−3^ M^6^. Three marine soil isolates (S3, S4 and K8) synthesized asparaginase with yield ranging from 24.6 to 49.2 IU/ml. Isolate S3 showed the highest productivity of 49.2 IU/ml and optimum activity at pH 7.5 and 50 °C. The Km and Vmax of partially purified enzyme were approximately 24 μM and 51 IU/ml, respectively^7^.

Literature reports indicated that the enzyme’s biochemical and kinetic properties varies with the genetic nature of the microbial strain used, suggesting the need to search for other L-asparaginase sources^8^. Actinomycetes are a good source for the production of L-asparaginase^9^. Among the actinomycetes, *Streptomyces* species are distributed widely in marine and terrestrial habitats^10^ and are of commercial interest due to their unique capacity to produce novel metabolites. Several *Streptomyces* species such as *Streptomyces gulbargensis*^11^, *Streptomyces olivaceus* NEAE-119^12^, *Streptomyces parvus* NEAE-95^13^ and *Streptomyces brollosae* sp. *nov*., NEAE-115^14^ have been explored for L-asparaginase production. There are also reports of L-asparaginase production from *Streptomyces noursei* MTCC 10469, isolated from marine sponge *Callyspongia diffusa*^15^ and *Streptomyces aurantiacus* isolated from mangroves of Bhitarkanika^16^.

Most of the microbial L-asparaginase is intracellular in nature except few which are secreted outside the cell^9^. Extracellular L-asparaginase is more advantageous than intracellular type because of higher accumulation of enzyme in culture broth under normal conditions, easy extraction and downstream processing^11^, the extracellular L-asparaginase in bacteria is protease deficient and the liberated protein exported to the medium is mostly soluble, biologically active and has an authentic N-terminus, relatively free from endotoxins those results in minimization of adverse effects. Secretion also facilitates proper folding of proteins specially that requiring disulfide bridge formation, as it passes through a more favorable redox potential in the periplasmic space.

The present investigation deals with isolation, production, purification and characterization of an extracellular L-asparaginase, under submerged fermentation from newly isolated actinomycetes *Streptomyces fradiae* NEAE-82.

## Results and Discussion

### Morphology and cultural characteristics of the isolate no. NEAE-82

**Figure 1:**
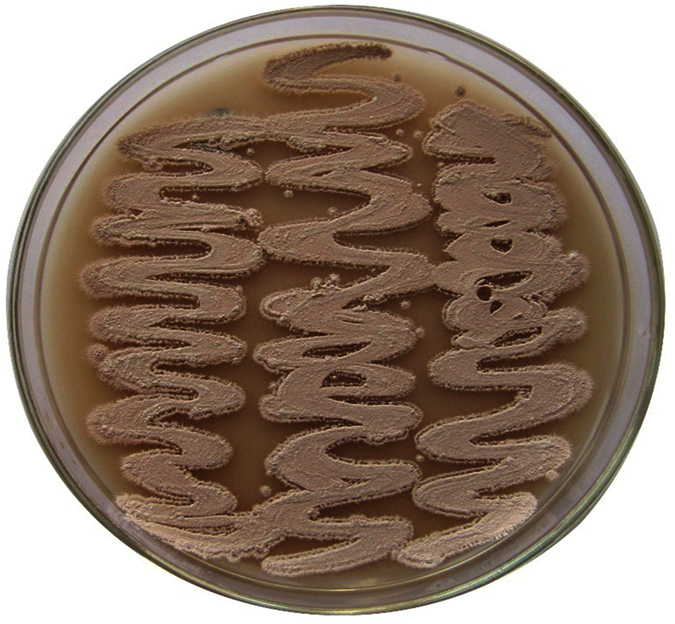
Color of the aerial mycelium of *Streptomyces* sp. NEAE-82 grown on starch -nitrate agar medium for 7–14 days of incubation at 30 °C.

**Figure 2:**
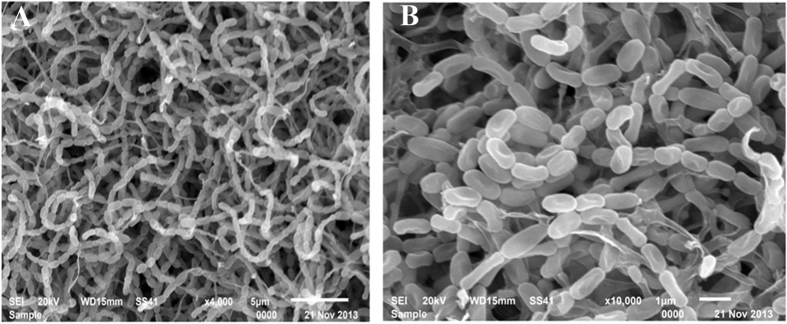
Scanning electron micrograph showing the spore-chain morphology and spore-surface ornamentation of strain NEAE-82 grown on starch nitrate agar medium for 14 days at 30 °C at magnification of 4000X (**A**) and 10000X (**B**).

The colonial morphology of a 14 day culture of strain NEAE-82 grown on yeast extract/malt extract agar (ISP 2 medium) revealed that strain NEAE-82 had the typical characteristics of the genus *Streptomyces*^17^. It is aerobic, mesophilic, Gram-positive actinomycete that develops abundant and well-developed substrate and aerial mycelium. Strain NEAE-82 produced reddish brown aerial mycelium (Fig. 1) on yeast extract-malt extract agar, oatmeal agar, inorganic salts-starch agar, and peptone-yeast extract iron agar. Not-distinctive aerial mycelium on glycerol-asparagine agar and tyrosine agar. Substrate mycelium with no distinctive pigments on most tested medium and brown substrate mycelium was produced on peptone-yeast extract iron agar (Table 1). Substrate mycelium pigment is not a pH indicator. No pigment found in medium in yeast extract -malt extract agar, oatmeal agar inorganic salt-starch agar, glycerol–asparagine agar or tyrosine agar, faint brown pigments formed in peptone-yeast extract iron agar. Melanoid pigments not formed in peptone-yeast extract iron agar and tyrosine agar. Strain NEAE-82 grew well on yeast extract -malt extract agar (ISP medium 2), oatmeal agar (ISP medium 3), inorganic salt-starch agar (ISP medium 4), peptone-yeast extract iron agar (ISP medium 6) but poor growth on glycerol–asparagine agar (ISP medium 5) and tyrosine agar (ISP medium 7). Strain NEAE-82 formed an extensively branched substrate mycelium and aerial hyphae which differentiated into long straight spore chains. Spore chains in section Retinaculiaperti including open spiral spore chains (Fig. 2). Flexuous or spiral spore chains are seen on starch-nitrate agar. Mature spore chains generally have 10–50 spores per chain; longer chains are sometimes observed. Spore surface is smooth. Verticils are not present. The mycelium does not fragment.

### Physiological characteristics

**Figure 3:**
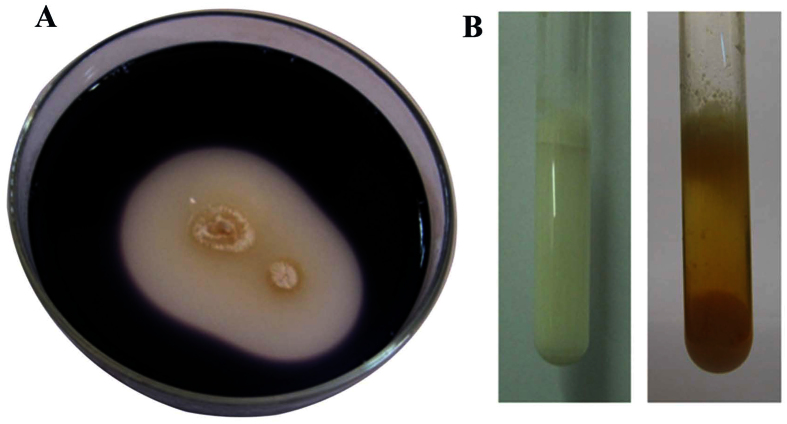
(**A**) Plate assay showing zone of hydrolysis of starch by strain NEAE 82. All the starch in the medium near the microbe has been hydrolyzed by extracellular amylases; (**B**) Coagulation and peptonization of milk by strain NEAE 82.

The physiological and biochemical reactions of strain NEAE-82 are shown in Table 2. It exhibited no antimicrobial activities against *Staphylococcus aureus, Alternaria solani and Bipolaris oryzae. Sacchromyces cerevisiae, Candida albicans, Bacillus subtilis, Escherichia coli, Pseudomonas aeruginosa, Rhizoctonia solani, Fusarium oxysporum, Aspergillus niger* and *Klebsiella pneumoniae.* α–amylase (starch hydrolysis) (Fig. 3), protease (degradation of casein), cellulase (growth on cellulose), chitosanase and L-asparaginase of strain NEAE-82 were produced. Melanin production, lecithinase activity and uricase were not produced. Coagulation and peptonization of milk (Fig. 3) and gelatine liquefaction were positive. Maximum NaCl tolerance was 6% (w/v). D-fructose, D-xylose, D-galactose, D-Glucose, L-arabinose, ribose, D-mannose, sucrose, maltose, cellulose and trehalose are utilized for growth, only traces of growth on rhamnose. The optimal growth temperature of strain NEAE-82 was 30 °C and optimal pH was 8.5.

### 16S rRNA gene sequence comparisons and phylogenetic analysis

**Figure 4:**
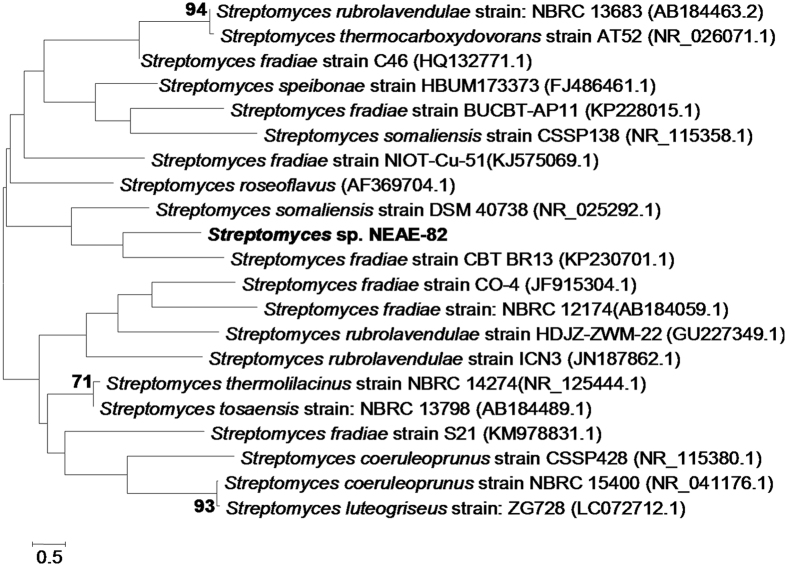
Neighbour-joining phylogenetic tree based on 16S rRNA gene sequences, showing the relationships between strain NEAE-82 and related species of the genus *Streptomyces*. Only bootstrap values above 50%, expressed as percentages of 1000 replications, are shown at the branch points. GenBank sequence accession numbers are indicated in parentheses after the strain names. Phylogenetic analyses were conducted in the software package MEGA4. Bar, 0.2 substitution per nucleotide position.

The 16S rRNA gene sequence (1508 bp) was determined for strain NEAE- 82. A BLAST search^18^ of the GenBank database using this sequence showed its similarity to that of many species of the genus *Streptomyces*. A phylogenetic tree (Fig. 4) based on 16S rRNA gene sequences of members of the genus *Streptomyces* was constructed according to the neighbour-joining method of Saitou and Nei^19^ with MEGA4^20^. This tree shows the close phylogenetic association of strain NEAE-82 with certain other *Streptomyces* species. Phylogenetic analysis indicated that the strain NEAE-82 consistently falls into a clade together with *Streptomyces somaliensis* strain DSM 40738 (GenBank/EMBL/DDBJ accession No. NR_025292.1) and *Streptomyces fradiae* strain CBT BR13 (GenBank/EMBL/DDBJ accession No. KP230701.1).

On the basis of the collected data and in view of the comparative study of the recorded properties of isolate No. NEAE-82 (Table 2) in relation to the closest related species of the genus *Streptomyces*, it is most closely related to the type strains of *Streptomyces fradiae* strain CBT BR13 (GenBank/EMBL/DDBJ accession No. KP230701.1) (99% sequence similarity)^21^. Therefore, this strain was identified as *Streptomyces fradiae* strain NEAE-82 and its sequencing product was deposited in the GenBank database under accession number KJ467538.

### Purification of L-asparaginase from *Streptomyces fradiae* NEAE-82

**Figure 5:**
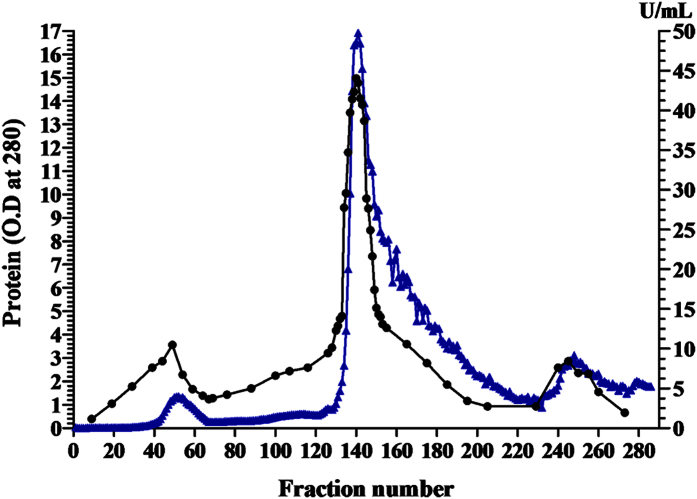
Purification of L-asparaginase produced by *Streptomyces fradiae* NEAE-82 using ion exchange on DEAE Sepharose CL-6B. (▲) refer to protein, (●) refer to L-asparaginase activity.

The crude culture filtrate of *Streptomyces fradiae* NEAE-82 had a total activity of 29507.688 U with protein content 3214.705 mg; the specific activity was 9.179 U/mg protein. The ammonium sulphate concentrated enzyme preparation had a protein content of 196.6 mg and the specific activity was 13.008 U/mg protein, showing purification fold of 1.417 with enzyme recovery at this step was 8.667 per cent. The fractions collected after ammonium sulphate precipitation were loaded on the column packed with DEAE Sepharose CL-6B and fractions of 2 ml were collected and analyzed for enzyme activity and protein content (Fig. 5). A total of 285 fractions were collected that showed one major L-asparaginase peak on the chromatogram. The total activity was 1442.393 with 47.081 mg protein after ion exchange chromatography through DEAE Sepharose CL-6B column. The specific activity of the purified enzyme was 30.636 U/mg of protein. Summary of the purification steps of the L-asparaginase produced by *Streptomyces fradiae* NEAE-82 is presented in Table 3.

In *Streptomyces albidoflavus*, L-asparaginase has been purified in CM Sephadex C-50 column up to 99.3 folds^9^. In another report, 106 folds purified L-asparaginase was obtained from *Pseudomonas aeroginosa* 50071^22^ by the final CM Sephedex C-50 column chromatography. Recently L-asparaginase purity of about 98.23 folds was reported in *Streptomyces noursei*^15^ and 82.12 folds was reported in *Streptomyces gulbergenesis*^11^ in final purification.

### Kinetics properties of the purified L-asparaginase

The activity of L-asparginase of *Streptomyces fradiae* NEAE-82 was evaluated at different levels of pH, temperature, effect of substrate concentration and incubation time.

### Effect of pH on L-asparaginase activity

**Figure 6:**
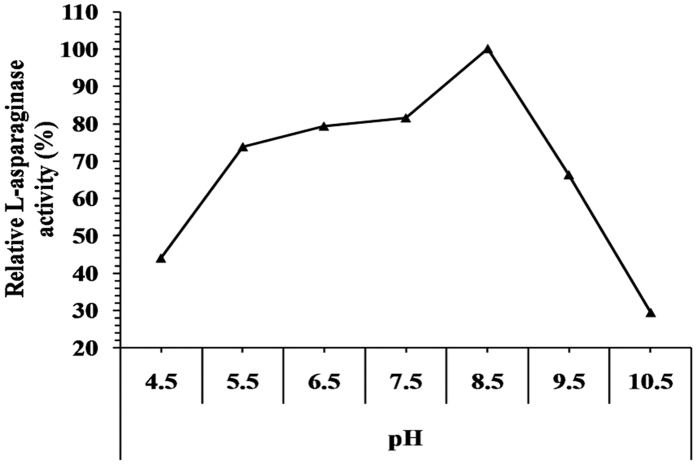
Activity of L-asparaginase as a function of the pH of the reaction.

L-asparaginase activity was studied as a function of pH in range between 4.5–10.5 (Fig. 6). L-asparaginase was active over broad pH ranges (4.5–10.5). The enzyme activity increase gradually till pH 8.5 with maximum activity 20.932 IU (relative activity, 100%). At higher pH’s, enzyme activity was decreased. The enzyme retains up to 29.348% of activity at pH 10.5 compare to 44.021% at pH 4.5. It is known that L-asparaginase can completely lose its activity and also, recover it partially, depending on the exposure conditions. These results coincide with that of Dhevagi and Poorani^23^ who reported the maximal L-asparaginase activity of *Streptomyces* sp. PDK7 was between pH 8.0 and 8.5, and the optimal L-asparaginase activity extracted from *Streptomyces gulbargensis* was 9.0^11^. L-asparaginase is one of the amidases that are generally active and stable at neutral and alkaline pH, whereas, pH 5.0 to 9.0 were reported earlier to be optimum for amidase activity^24^. L-asparaginase, purified from *Streptomyces acrimycini* NGP, exhibited maximum activity at pH 7.0^25^; membrane bound L-asparaginase from *Tetrahymena pyriformis* acts optimally at pH 9.6^26^ and the optimal L-asparaginase activity from *Corynebacterium glutamicum* was reported at pH 7.0^27^. Maximum activity of purified L-asparaginase occurred at pH 9 was obtained for *Pseudomonas aeruginosa* 50071^22^.

### Effect of temperature on L-asparaginase activity

**Figure 7:**
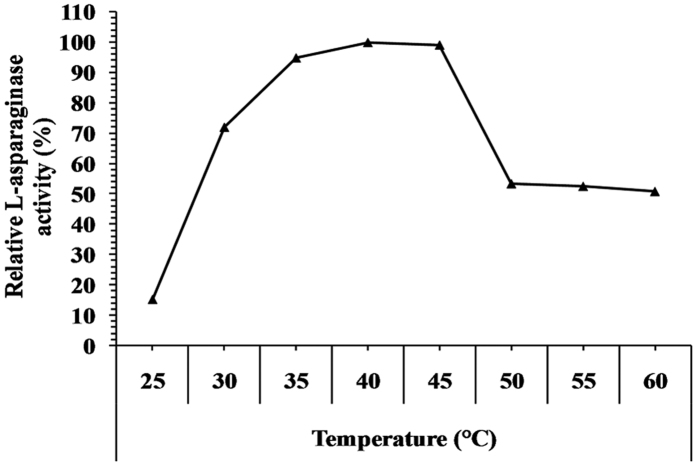
Effect of the temperature on L-asparaginase activity.

The temperature optimum of L-asparaginase from *Streptomyces fradiae* NEAE-82 is shown in Fig. 7. It was active at wide range of temperature condition from 25–60 °C. The maximum L-asparaginase activity of 26.848 IU was obtained at 40 °C. At higher temperature the L-asparaginase activity declined. The enzyme retains 50.85% of its activity at 60 °C. Our results were in agreement with a previous study which reported that the maximum activity of L-asparaginase purified from *Streptomyces gulbargensis* was at 40 °C^11^. Manna *et al*.^28^ have found 37 °C to be the optimum temperature for the enzyme activity obtained from *Pseudomonas stutzeri* MB-405. However, optimum temperature for L-asparaginase activity obtained from *Erwinia* sp. showed maximum activity at 35 °C^29^.

### Effect of substrate concentration on the activity of L-asparaginase

**Figure 8:**
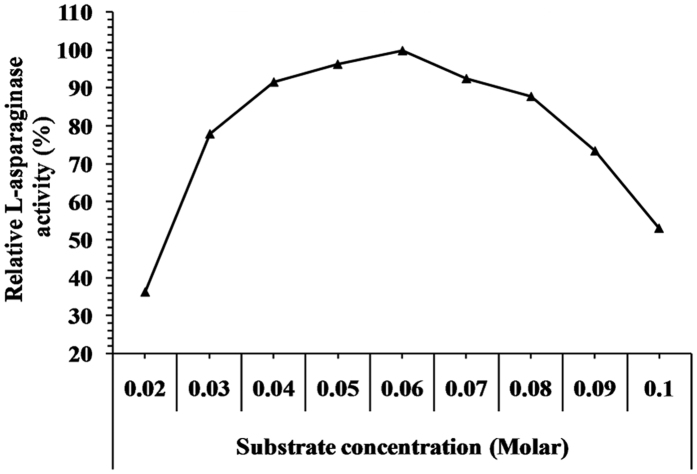
Effect of the substrate concentration of the reaction on L-asparaginase activity.

In this experiment, the influence of substrate concentration on L-asparaginase activity was examined by using different concentration of substrate ranging from 0.02 to 0.1 Molar to determine the optimum concentration of substrate required to give the highest L-asparaginase activity. The results in Fig. 8 showed a gradual increase in the enzyme activity with the increase in substrate concentration from 0.02 to 0.06 Molar. However, further increase in substrate concentration (0.07–0.1 Molar) lead to decrease in enzyme activity to 53.03% with 0.1 Molar substrate. The optimum substrate concentration for L-asparaginase activity was observed at 0.06 Molar.

**Figure 9:**
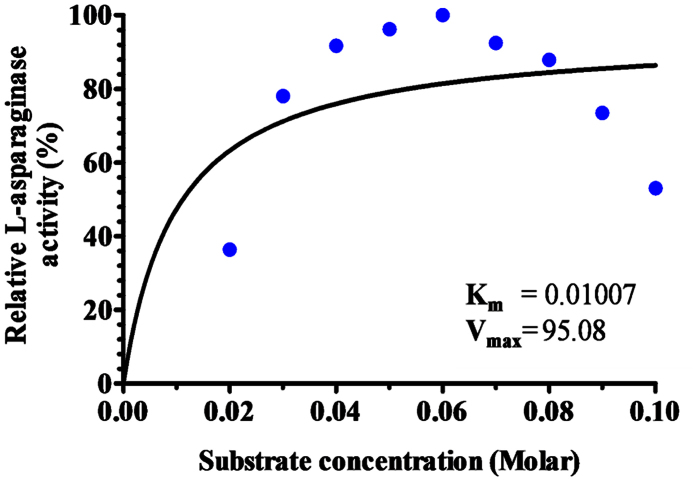
Michaelis-Menten plot for L-asparaginase produced by *Streptomyces fradiae* NEAE-82.

The typical Michaelis-Menten relationship was obtained between the substrate concentrations and the initial velocity of the reaction. Michaelis-Menten plot showed in Fig. 9 illustrated the K_m_ and V_max_ values for L-asparaginase enzyme. The plot gave K_m_ value of 0.01007 M and V_max_ of 95.08 Uml^−1^min^−1^ for the hydrolysis of L-asparagine. K_m_ value is defined as the substrate concentrations that result in half maximal velocity for the enzymatic reaction, or an equivalent way of stating substrate concentration at which half of the enzyme active sites in the sample are filled (i.e. saturated) by substrate molecules in the steady state. It can be used as a relative measure of substrate affinity with the studied enzyme. K_m_ reflects the affinity of the enzyme for its substrate^30^. The lower the K_m_ is the stronger binding ability the enzyme has. V_max_ is the limiting velocity as substrate concentrations get very large. V_max_ is expressed in units of product formed per unit of time. If the molar concentration of enzyme is known, V_max_ be expressed as moles of product formed per second per mole of enzyme sites^31^. This is the turnover number, the number of molecules of substrate converted to product by one enzyme site per second. However, there are many factors that affect on kinetic parameters of the enzymes (K_m_ and V_max_) such as; type of enzyme, different forms of enzyme (crude, modified or purified), changes in enzyme conditions (pH, temperature, etc.), source of the enzyme (different microorganisms), type of used substrates, and the assay procedures^32^.

### Effect of incubation time on enzyme activity

**Figure 10:**
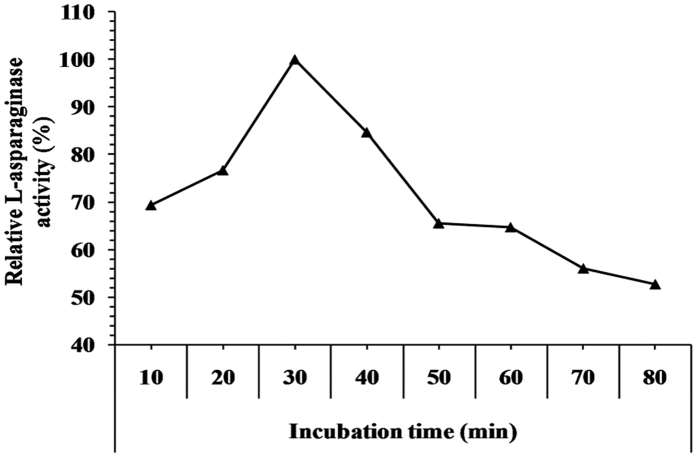
Effect of different incubation periods on L-asparaginase activity.

The L-asparaginase activity (Fig. 10), increased as the incubation time increased up to 30 min (L-asparaginase activity of 34.128 IU). After which only a slight decrease in L-asparaginase activity was observed. El-Bessoumy *et al*.^22^ reported that, maximum activity of L-asparaginase purified from *Pseudomonas aeruginosa* 50071 was at 30 min. In addition, the effect of incubation time on the activity of purified L-asparaginase from *Streptomyces noursei* showed that the activity reached its maximum at 35 min^15^. The decrease in L-asparaginase activity was observed after longer period of incubation with substrate. The decrease may due to the product inhibition.

### Thermal stability

**Figure 11:**
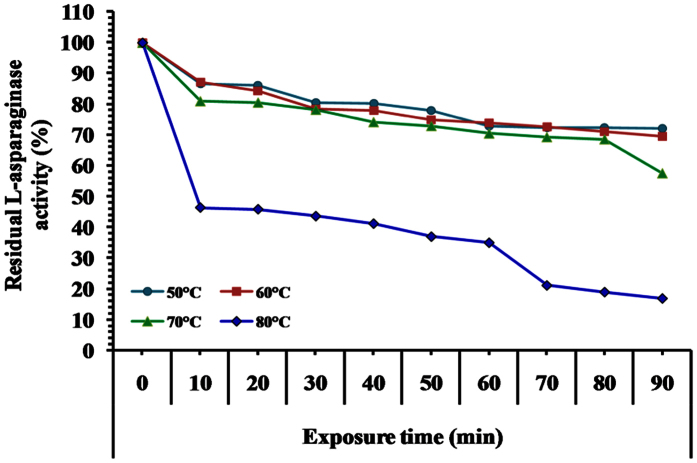
Thermal stability of L-asparaginase as a function of the time of the reaction.

The effect of temperature on the stability of L-asparaginase showed maximum enzyme activity at 50 °C (Fig. 11). Around 86.126% of the initial activity was retained by the enzyme after 20 min of incubation at 50 °C. About 72.072% of L-asparaginase activity was lost after incubation at 50 °C for 90 min, while a rapid decrease in the enzyme activity (16.874%) was observed after incubation at 80 °C for 90 min. Thermal inactivation process of the enzyme follows the theoretical curve of a sample first order reaction. However, linear regression of the obtained data was assayed to determine half life time (T_1/2_) as shown in Table 4. The half-life time (T_1/2_) was 184.91 min at 50 °С, while being 179.53 min at 60 °С. On the other hand, destruction of enzyme activity was observed at 80 °C with low half-life time (75.36 min). It can be concluded from the previous results that the higher thermal stability behavior of L-asparaginase was at 50 °C. Results presented in Table 4 were normalized to activity of un-incubated enzyme (time 0 = 100%) for the percentage of activity remaining. Heat inactivation half-life (T_1/2_) and heat deactivation constant (*k*) were determined by fitting the data to a first-order decay curve using Graph-Pad Prism software. An earlier study reported no significant loss of L-asparaginase activity purified from *Streptomyces radiopugnans* MS1, when the enzyme was pre-incubated at 40 °C for 60 min^33^. Similar results were recorded with *Streptomyces noursei*^15^, *E. carotovora*^34^, *Pseudomonas stutzeri* MB 405^28^.

A source-dependent variation of physicochemical and biochemical properties, like optimum pH, temperature, substrate specificity, inhibition pattern, etc., of microbial enzymes is well documented^8^. Comparative evaluation of L-asparaginase for its potential activity from different microbial sources revealed that biochemical and therapeutic properties differ with source of strain in addition to enzyme properties. L-asparaginases from *Erwinia chrysanthemi* and *E. coli* are currently in clinical use as effective drugs in the treatment of the acute lymphoblastic leukemia. Comparison of the two enzymes, lead to the conclusion that most patients allergic to the *E. coli*- L-asparaginase, *Erwinia* asparaginase is considered less toxic and is frequently employed compared with allergic reactions to *E. coli*- L-asparaginase. However, *Erwinia* asparaginase had a shorter half life than *E. coli*^4^. The optimum temperature for L-asparaginase activity obtained from *E. coli* showed maximum activity at 37 °C and pH 7–8^35^. The maximum L-asparaginase activity obtained from *Erwinia aroideae*^36^ occurred between pH 7 and 8 and optimum temperature for L-asparaginase activity obtained from *Erwinia* sp. showed maximum activity at 35 °C^29^. However, the characterization of the enzyme produced by newly isolated actinomycetes *Streptomyces fradiae* NEAE-82 revealed that the optimum pH, temperature and incubation time for maximum L-asparaginase activity were found to be 8.5, 40 °C and 30 min, respectively. It was active at wide range of temperature condition from 25–60 °C and active over broad pH ranges (4.5–10.5). The physiological pH is one of the perquisites for antitumor activity^37^ under alkaline pH condition, L-asparaginase becomes a competitive inhibitor^38^. This property of the enzyme is most suitable for complete elimination of asparagines from the body when tumor patient is treated with L-asparaginase *in-vivo* and clarified that the enzyme produced by *Streptomyces fradiae* NEAE-82 under the present study (optimum pH 8.5) has effective antitumor activity.

### Molecular weight determination by Sodium Dodecyl Sulphate- Polyacrylamide Gel Electrophoresis (SDS–PAGE)

**Figure 12:**
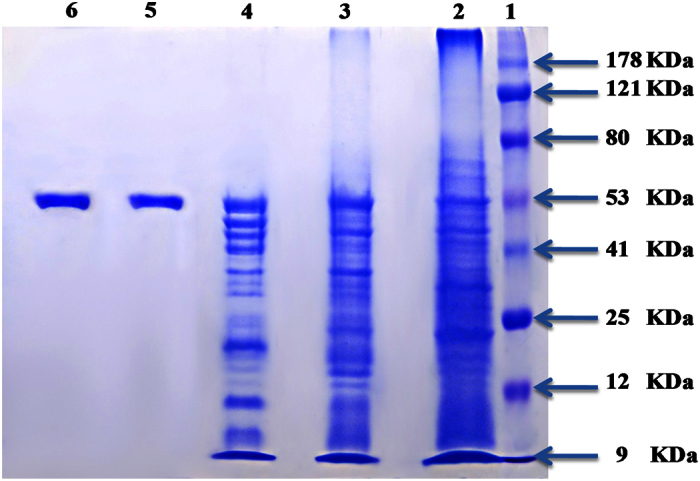
SDS-polyacrylamide gel electrophoresis of the purified L-asparaginase from *Streptomyces fradiae* NEAE-82. Lane 1: Protein marker; Lane 2–4: Ammonium sulphate fractions (50, 60, 70% respectively); Lane 5, 6: Purified protein.

The molecular weight of the extracted enzyme was determined by performing SDS-PAGE (sodium dodecyl sulfate–polyacrylamide gel electrophoresis) according to the method of Laemmli^39^, with a separating acrylamide gel of 10% and stacking gel 5% containing 0.1% SDS. After the electrophoresis, the gel was stained in 0.025 Coomassie brilliant blue R-250 and destained with a solution of methanol- acetic acid and water in the ratio of 4:1:5. The molecular weight of the purified L-asparaginase was determined in comparison with standard molecular weight markers (molecular mass range: 9–178 kDa). SDS–PAGE of the enzyme preparation revealed only a single distinctive protein band for the pure preparation of L-asparaginase with an apparent molecular weight of 53 kDa (Fig. 12).

L-asparaginase is known as a homotetramer. Four active sites are located at the interface between two subunits forming an intimate dimer, and hence the asparaginase is more accurately described as a dimer of dimers with a molecular mass of approximately 120 to 160 kDa possessing antitumor activity^40^. The molecular weight of the L-asparaginase was found to be varied according to the source of enzyme like 80 kDa in *Corynebacterium glutamicum*^27^, 140 kDa in *Streptomyces* sp. PDK2^23^ and 116 kDa in *S*. *albidoflavus*^9^. Purified L-asparaginase from *S*. *tendae* checked by SDS-PAGE revealed a distinct protein band near 97.4 kDa^41^. In this respect, L-asparaginases purified from *Pseudomonas stutzeir* MB-405, *Thermus thermophilus* and *Escherichia coli* were with smaller molecular weight values ranging from 33–34 kDa^28^,^42^. Other reports on production and purification of L-asparaginase from *E. coli* revealed that the molecular weight was determined as 153 KDa with the help of SDS-PAGE^43^. The functional L-asparaginase from *E. coli* is a homotetramer with a molecular weight of about 142 kDa^44^. Purified L-asparaginase from *Streptomyces gulbargensis*^11^, *Streptomyces albidoflavus*^9^, *Streptomyces* PDK2^23^ and *Streptomyces noursei*^15^ exhibited a molecular weight of 85, 112, 140 kDa and 102 kDa, respectively. Reports on production and purification of L-asparaginase from *Pseudomonas aeruginosa* 50071 by SDS- PAGE revealed a peptide chain with molecular weight of 160 kDa^22^.

### Cytotoxicity and anticancer activities of L-asparaginase on non-cancerous and cancer cells

**Figure 13:**
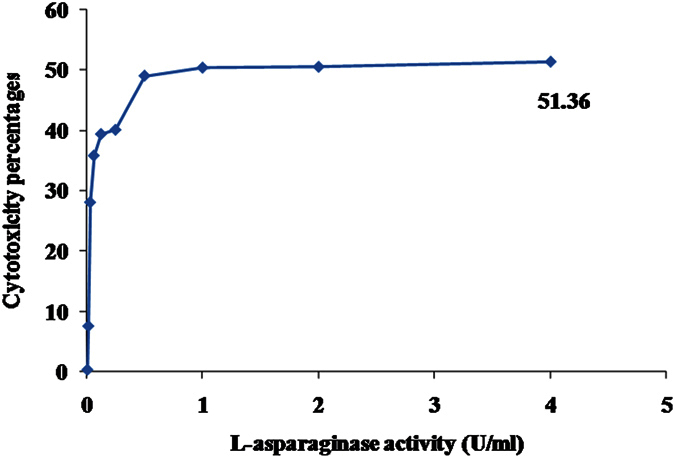
Cytotoxicity and safety assay of the purified L-asparaginase on non-cancerous human fibroblast cells.

**Figure 14:**
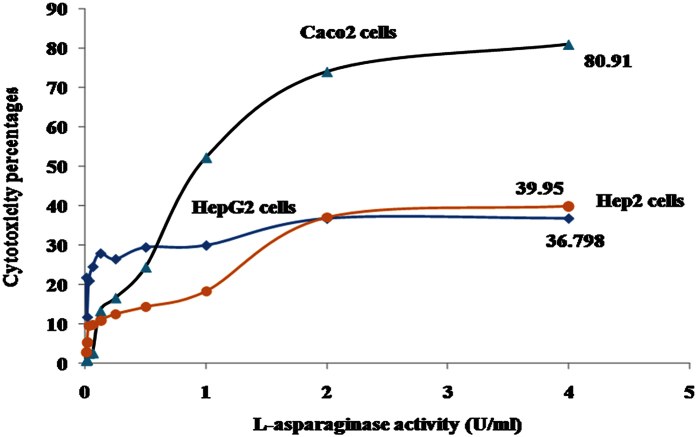
The anticancer effects of the purified L-asparaginase on Caco2, Hep2 and HepG2 cells.

**Figure 15:**
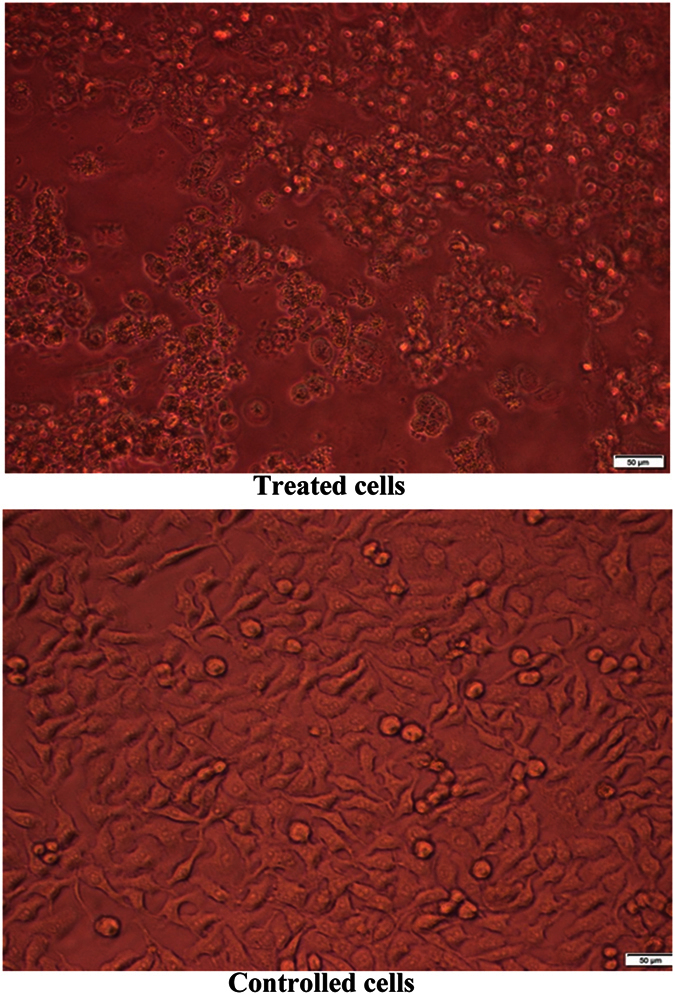
The anticancer effect of the purified L-asparaginase on Caco2 cells after 48 of treatment, cells undergoing apoptosis are characterized by cellular rounding up, shrinkage, membrane blebbing and loss of cell adhesion.

The safety pattern of purified L-asparaginase was scanned on human fibroblast cells. Generally, the obtained data revealed that, the treatment IC_50_ on all cells ranged from 2 to 4 U/ml (Fig. 13). The anti-proliferative activity of L-asparaginase on cancer cells was quantitatively estimated on HepG2, Hep2 and Caco2 cells. The obtained data generally indicated that, the activity of the extract against Caco2 was superior to that with either HepG2 or Hep2 cells (Figs 14 and 15, Table 5). Up on cancer cells treatment, the selected recommended dose (4 U/ml) showed anti-proliferation activities against Caco2 cells with percentages 80.9 with cancer cell selectivity index reached 4. On the other hand, the anticancer activity of the purified on Hep2 and Caco2 cells reached 39.95 and 36.79%, respectively with selectivity index 0.88 and 0.64, respectively. Sialyl Lewis^X^ (sLe^x^) is a tetrasaccharide carbohydrate that is often linked to cell-surface glycoproteins. Sialic acids in general play important roles in biological characteristics of cancer and other cells because it overexpressed in some tumor cell types and that are implicated in cellular invasiveness, differentiation and tumourigenecity^45^,^46^. Sialyl Le^x^ is a major E-selectin ligand, mediating both homotypic and heterotypic cell adhesion of endothelial cells^47^. Endothelial cell surface E-selectin was shown to be necessary for endothelial cell (EC) binding to colon cancer cells^47^. L-asparaginase has been reported to prevent glycosylation several forms as sialylation, of newly synthesized proteins^48^. So, we suggest that as almost the same reports of Yu *et al*.^49^ in ovarian cancer, L-asparaginase could significantly alter the interactions between microvascular ECs, colon cancer cells, and ECM components, resulting incolon cancer cell injury.

## Materials and Methods

### Microorganisms and cultural conditions

*Streptomyces* spp. used in this study were isolated from various soil samples collected from different localities of Egypt and Saudi Arabia. Actinomycetes had been isolated from the soil using standard dilution plate method procedure on Petri plates containing starch nitrate agar medium of the following composition (g/L): Starch, 20; KNO_3_, 2; K_2_HPO_4_, 1; MgSO_4_.7H_2_O, 0.5; NaCl, 0.5; CaCO_3_, 3; FeSO_4_.7H_2_O, 0.01; agar, 20 and distilled water up to 1 L; then plates were incubated for a period of 7 days at 30 °C. *Streptomyces* isolates were purified and maintained as spore suspensions in 20% (v/v) glycerol at −20 °C for subsequent investigation.

### Screening of L-asparaginase production by plate assay

It is generally observed that L-asparaginase production is accompanied by an increase in pH of the culture filtrates^50^. The plate assay was based on Gulati *et al*.^51^ method with the incorporation of pH indicator phenol red (prepared in ethanol) in medium containing L-asparagine (sole nitrogen source). Phenol red at acidic pH is yellow and at alkaline pH turns pink, thus a pink zone is formed around microbial colonies producing L-asparaginase. Screening of potential L-asparaginase producing actinomycetes was carried out with the use of asparagine dextrose salts agar (ADS Agar) (asparagine 1.0%, dextrose 0.2%, K_2_HPO_4_ 0.1%, MgSO_4_ 0.05%, agar 1.5%), pH was adjusted to 6.8 and supplemented with phenol red as a pH indicator (0.009% final concentration)^52^ and sterilized at 1.5 atmospheric pressure for 20 min. Inoculated plates were incubated at 30 °C for 7 days. Plates were examined for change in color of medium from yellowish to pink due to change of pH indicating the positive asparaginase activity. Colonies with pink zones were considered as L-asparaginase-producing strains. Isolates exhibiting L-asparaginase activity were selected for further study. Control plates were prepared as uninoculated medium and medium without dye.

### Inoculum preparation

250 ml Erlenmeyer flasks containing 50 ml of asparagine dextrose salts broth (L-asparagine 1.0%, dextrose 0.2%, K_2_HPO_4_ 0.1%, MgSO_4_ 0.05%) were inoculated with three disks of 8 mm diameter taken from the 7 days old stock culture grown on starch nitrate agar medium. The flasks were incubated for 48–72 h in a rotatory incubator shaker at 30 °C and 150 rpm and were used as inoculum for subsequent experiments.

### Production of L-asparaginase by submerged fermentation

The selected strain was cultured in fifty ml of asparagine dextrose salts broth medium (at a specified pH) dispensed in 250 ml Erlenmeyer conical flasks. The inoculated flasks were incubated on a rotatory incubator shaker at 30–37 °C with shaking at 150–250 rpm. After the specified incubation time for each set of experimental trials, the mycelium of the tested isolate was collected by centrifugation at 5000 × *g* for 30 min at 4 °C.

### Assay of L-asparaginase activity

L-asparaginase activity was determined by measuring the amount of ammonia released by nesslerization according to the method described by Wriston and Yellin^52^. The reaction mixture containing 1.5 ml of 0.04 M L-asparagine prepared in 0.05 M Tris-HCl buffer, pH 8.6 and 0.5 ml of an enzyme to make up the total volume to 2 ml. The tubes were incubated at 37 °C for 30 minutes. The reaction was stopped by adding 0.5 ml of 1.5 M trichloroacetic acid (TCA). The blank was prepared by adding enzyme after the addition of TCA. The precipitated protein was removed by centrifugation at 10,000 × *g* for 5 min and the liberated ammonia in the supernatant was determined colorimetrically by direct nesslerization by adding 1 ml Nessler’s reagent into tubes containing 0.5 ml of clear supernatant and 7 ml distilled water and incubated at room temperature for 20 min. A yellow coloration indicates the presence of ammonia: at higher concentrations, a brown precipitate may form. The yellow color was read using a UV-visible spectrophotometer (Optizen Pop –UV/Vis spectrophotometer) at 480 nm. The amount of ammonia liberated was calculated using ammonium (ammonium chloride) standard curve. One unit (U) of L-asparaginase is defined as the amount of enzyme which catalyzed the formation of 1 μmole of ammonia from L-asparagine per minute at 37 °C and pH 8.6. The enzyme activity was expressed in terms of units per gram dry fermented substrate (U/gds).

### Morphology and cultural characteristics

The morphology of the spore chain and the spore surface ornamentation of strain NEAE-82 were examined on inorganic salt/starch agar after 14 days at 30 °C. The gold-coated dehydrated specimen can be examined at different magnifications with Analytical Scanning Electron Microscope Jeol JSM-6360 LA operating at 20 Kv at the Central Laboratory, City for Scientific Research and Technological Applications, Alexandria, Egypt. Aerial spore-mass color, substrate mycelial pigmentation and the production of diffusible pigments were observed on ISP medium 1–7 as described by Shirling and Gottlieb^53^.

### Physiological characteristics

Physiological characteristics were performed following the methods of Shirling and Gottlieb^53^. The ability of the organism to inhibit the growth of four bacterial (*Pseudomonas aeruginosa*, *Staphylococcus aureus*, *Escherichia coli*, or *Klebsiella*), and five fungal strains (*Rhizoctonia solani*, *Alternaria solani*, *Bipolaris oryzae*, *Fusarium oxysporum*, *Fusarium solani*) was determined.

### 16S rRNA sequencing

The PCR amplification reaction and sequencing were performed in accordance with the methods described by El-Naggar *et al*.^12^. Sequencing product was deposited in the GenBank database under accession number KJ467538.

### Sequence alignment and phylogenetic analysis

The partial 16S rRNA gene sequence of strain NEAE-82 was aligned with the corresponding 16S rRNA sequences of the type strains of representative members of the genus *Streptomyces* retrieved from the GenBank, EMBL, DDBJ and PDB databases by using BLAST program (www.ncbi.nlm.nih.gov/blst)^18^ and the software package MEGA4 version 2.1^20^ was used for multiple alignment and phylogenetic analysis. The phylogenetic tree was constructed via the neighbor-joining algorithm^19^ based on the 16S rRNA gene sequences of strain NEAE-82 and related organisms.

### Purification of L-asparaginase from *Streptomyces fradiae* NEAE-82

All purification steps were carried out at 4 °C using crude enzyme extract. The extracellular crude enzyme was prepared at the end of the fermentation period by centrifugation at 11,000 × *g* for 30 min. The cell free supernatant was used as the crude enzyme preparation. Finely powdered ammonium sulfate was slowly added to the clear supernatant obtained after centrifugation to reach 45% saturation and incubated overnight. The precipitate was collected by centrifugation at 11000 × *g* for 30 min, while the supernatant was brought to 55–85% saturation with ammonium sulphate. Then the precipitates were collected separately by centrifugation, dissolved in a minimal amount of 50 mM Tris-HCl buffer pH 8.4 and dialyzed overnight against the same buffer. After dialysis, the samples were used for protein estimation and enzyme assay by the method of Lowry *et al*.^54^ and direct Nesslerization method^52^, respectively and stored at 4 °C for further purification.

The dialyzed enzyme solution obtained from the previous step was loaded into a diethylaminoethyl (DEAE) Sepharose CL-6B column (2.3 cm × 12 cm) that was pre-equilibrated with 50 mM Tris-HCl buffer (pH 8.4). The column was washed with two column volume of the above buffer and the adsorbed protein was isocratic eluted using a 0.5 M NaCl in 50 mM Tris–HCl (pH 8.4). Fractions were collected at a flow rate of 1 ml min^−1^ (each fraction containing 2 ml) using a microfractionator (Bio-Red). All chromatographic runs were monitored for protein at 280 nm. The fraction were collected and examined for enzyme activity and protein content by procedures described earlier^52^,^54^. Fractions showing high L-asparaginase activity were collected for further use.

### Characterization of L-asparaginase enzyme

The effect of the incubation time on L-asparginase activity was studied by incubating the reaction mixture for different times (10, 20, 30, 40, 50, 60, 70 and 80 min), then the activity of the enzyme was determined. The optimum pH of L-asparaginase activity was studied; L-asparaginase enzyme was pre-incubated with 0.05 M buffers over a range of pH 4–10 under assay conditions, and the amount of ammonia liberated was determined. The buffers used were citric acid- Na_2_HPO_4_ (pH 4.5–7.5), Tris-HCl (pH 8.5), and glycine-NaOH (pH 9.5–10.5). The optimum temperature for L-asparaginase activity was determined by incubating the assay mixture at different temperatures ranging from 25 to 60 °C in 0.05 M Tris-HCl buffer under assay conditions. Effect of different substrate concentration on L-asparaginase activity was determined by incubating 0.1 ml from the purified enzyme with different concentration of the specific substrate (0.02, 0.03, 0.04, 0.05, 0.06, 0.07, 0.08, and 1 M) and then the activity of the enzyme was determined under assay conditions.

### Determination of kinetic properties (K_m_, V_max_)

The reaction kinetics of the purified enzyme was determined from Lineweaver-Burk plots^55^ with L-asparagine as substrate under defined assay conditions. The Michaelis–Menten constant (K_m_) and maximal velocity (V_max_) were determined for the enzyme at each of the measured temperatures using the Michaelis–Menten equation:


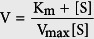


where V is the reaction velocity (a function of enzyme concentration), [S] is the substrate concentration, K_m_ is the substrate concentration at half-maximal velocity, and V_max_ is the maximal velocity. V_max_ and K_m_ values were determined using nonlinear regression^56^.

### Enzyme thermal stability

Thermal stability of the L-asparaginase was carried by pre-incubating the buffered enzyme prepared in absence of its substrate for different time interval ranging from 0.0 to 90 min at different temperatures (50, 60, 70 and 80 °С). After incubation, the enzyme was cooled then the residual activities were assayed under the standard conditions.

### Molecular weight determination and checking of enzyme homogeneity

The molecular weight of the subunit and the purity of enzyme was checked by performing sodium dodecyl sulfate–polyacrylamide gel electrophoresis (SDS-PAGE). SDS-PAGE was performed following the method of Laemmli^39^, with separating acrylamide gel 10% (w/v) and stacking gel 5% (w/v) containing 0.1% (w/v) SDS. After the electrophoresis, the gel was stained with coomassie brilliant blue R-250 and de-stained with a solution of methanol, acetic acid and water in the ratio of 4:1:5. Approximate subunit molecular weight of L-asparaginase was determined using standard wide range molecular weight marker (9–178 kDa).

### Cell lines

#### Cancerous cells

Human epithelial colorectal adenocarcinoma cells (caco2 cells), liver hepatocellular carcinoma cells (HepG2 cells) and human laryngeal carcinoma cells (Hep2 cells).

### Non-cancerous cells

Human fibroblast cells (FB).

### Cytotoxicity and anticancer assay

In order to determine both safety and the anticancer activities of the purified L-asparaginase on both cancerous (CaCo2, HepG2 and Hep2 cells) and non-cancerous cells (Human fibroblast cells), neutral red assay protocol was used. Briefly, 100 *μ*l of each of serially diluted purified enzymes in either RPMI or DMEM media were incubated with about 6 × 10^4^ cell/ml of each cell type on 96-well plates. After 48 hours, the cellular cytotoxic effects were quantified using neutral red assay^57^.

### Selectivity index (SI)

The degree of anticancer selectivity of the tested enzyme was expressed as the previous report^58^ with a minor modification^59^, SI = LC50 of pure compound in a normal cell line/LC50 of the same pure compound in cancer cell line, where LC50 is the concentration required to kill 50% of the cell population.

## Conclusion

Production of L-asparaginase using different microbial systems has attracted much attention, owing to the cost-effective and ecofriendly nature. The present study revealed an efficient production of an extracellular L-asparaginase throughout various purification steps from isolate *Streptomyces fradiae* NEAE-82 under submerged fermentation. From the culture filtrate of this strain, L-asparaginase enzyme was purified by column chromatography and molecular weight was determined by SDS-PAGE. The enzyme substrate specificity, kinetic parameters were also determined. Furthermore, high catalytic activity of the enzyme over a wide range of pH and temperature, and its considerable stability, makes it highly favorable for use as a potent anticancer agent, and for other applications in healthcare industry.

## Additional Information

**How to cite this article**: El-Naggar, N. E. *et al*. Purification, characterization, cytotoxicity and anticancer activities of L-asparaginase, anti-colon cancer protein, from the newly isolated alkaliphilic *Streptomyces fradiae* NEAE-82. *Sci. Rep.* **6**, 32926; doi: 10.1038/srep32926 (2016).

## Figures and Tables

**Table 1:** Culture characteristics of the *Streptomyces* sp. strain NEAE-82.

Medium	Aerial mycelium (spore-colour en masse)	Substrate mycelium	Diffusible pigment	Growth
ISP medium 2 (Yeast extract -malt extract agar)	Reddish brown	Not-distinctive	Non-pigmented	Excellent
ISP medium 3 (Oatmeal agar)	Reddish brown	Not-distinctive	Non-pigmented	Excellent
ISP medium 4 (Inorganic salt-starch agar)	Reddish brown	Not-distinctive	Non-pigmented	Excellent
ISP medium 5 (Glycerol asparagines agar)	Not-distinctive	Not-distinctive	Non-pigmented	Weak
ISP medium 6 (Peptone-yeast extract iron agar)	Reddish brown	Brown	Faint brown	Excellent
ISP medium 7 (Tyrosine agar)	Not-distinctive	Not-distinctive	Non-pigmented	Weak

The substrate mycelium pigment was not pH sensitive when tested with 0.05 N NaOH or 0.05 N HCl.

The diffusible pigment was not pH sensitive when tested with 0.05 N NaOH or 0.05 N HCl.

**Table 2:** Phenotypic properties that separate strain *Streptomyces* NEAE-82 from related *Streptomyces* species.

Characteristic	*Streptomyces* sp. strain NEAE-82	*Streptomyces fradiae*
Aerial mycelium on ISP mdeium 2	Reddish brown	Red
Substrate mycelium on ISP medium 2	Not-distinctive	Not-distinctive
Production of diffusible pigment	No diffusible pigment	No diffusible pigment
Spore chain morphology	Retinaculiaperti including open spirals	Retinaculiaperti morphology, including open spirals
Spore surface	Smooth	Smooth
Spore shape	Oval to oblong	
Melanin production on peptone-yeast extract iron agar (ISP medium 6)	−	−
Melanin production on tyrosine agar (ISP medium 7)	−	−
Max NaCl tolerance (%, w/v)	6	
Utilization of carbon sources (1%, w/v)		
D(−) Fructose	+	+
D(+) Xylose	+	±
D(+) Galactose	+	
D(+) Glucose	+	+
L-arabinose	+	+
Ribose	+	
D(+) Mannose	+	
Sucrose	+	±
Maltose	+	
Rhamnose	+	±
Raffinose	±	±
Cellulose	+	
Trehalose	+	

Data for reference species were taken from Bergey’s Manual^®^ of Systematic Bacteriology -volume five the actinobacteria (Goodfellow *et al*.^21^).

Abbreviations: +, Positive; −, Negative; ±, Doubtful; Blank cells, no data available.

The optimal growth temperature was 30 °C and optimal pH was 8.5. It exhibited no antimicrobial activities against *Staphylococcus aureus, Alternaria solani, Bipolaris oryzae, Sacchromyces cerevisiae, Candida albicans, Bacillus subtilis, Escherichia coli, Pseudomonas aeruginosa, Rhizoctonia solani, Fusarium oxysporum, Aspergillus niger* and *Klebsiella pneumoniae*. α–amylase (starch hydrolysis), protease (degradation of casein), cellulase (growth on cellulose), chitosanase and L-asparaginase of strain NEAE-82 were produced while uricase, lecithinase were not produced. Coaggulation and peptonization of milk and gelatin liquefaction were positive.

**Table 3:** Summary of the purification steps of the L-asparaginase produced by *Streptomyces fradiae* NEAE-82.

Purification step	Total protein content (mg)	L-asparaginase activity			
		Total activity (U)	Specific activity (U/mg protein)	Recovery (%)	Purification fold
Culture filtrate	3214.705	29507.688	9.179	100	1
(NH_4_)_2_SO_4_, post dialysis	196.6	2557.325	13.008	8.667	1.417
Ion exchange on DEAE Sepharose CL-6B	47.081	1442.393	30.636	4.888	3.338

**Table 4:** Half life time (T_1/2_) and heat deactivation constant (k) of L-asparaginase produced by *Streptomyces fradiae* NEAE-82.

**Half life time (min)**	
50 °C	184.91
60 °C	179.53
70 °C	150.29
80 °C	75.36
Thermal inactivation rate constant *k*_*d*_*1*(min^−1^)*	
50 °C	0.0037
60 °C	0.0039
70 °C	0.0046
80 °C	0.0092
**D-value (min)**	
50 °C	332.84
60 °C	323.16
70 °C	270.51
80 °C	135.64
Thermal inactivation rate constant *k*_*d*_ *2*(min^−1^)**	
50 °C	0.0021
60 °C	0.0021
70 °C	0.0026
80 °C	0.0051

*k_*d*_*1* is the deactivation constant after losing 50% of initial activity (at T_1/2_).

**k_*d*_*2* is the deactivation constant after losing 90% of initial activity (at D value).

**Table 5:** The Cancer cell selectivity index of the purified L-asparaginase.

Cell type	Cancer cells selectivity index (SI)
HepG2 cells	0.6472
Caco2 cells	4
Hep2 cells	0.8843
